# Recent advance in FGF23 – clinical perspectives

**DOI:** 10.1186/1687-9856-2015-S1-O9

**Published:** 2015-04-28

**Authors:** Toshimi Michigami

**Affiliations:** 1 Osaka Medical Center and Research Institute for Maternal and Child Health, Osaka, Japan

## 

Fibroblast growth factor 23 (FGF23) is a circulating factor that plays a central role in the renal reabsorption of Pi and metabolism of vitamin D. It is mainly produced by osteocytes in bone and exerts its effects on distant organs such as the kidney and parathyroid in an endocrine fashion. FGF23 increases renal Pi excretion by reducing the expression of type 2a and 2c sodium/phosphate (Na^+^/Pi) co-transporters in the proximal tubules. In addition, it decreases the renal production of 1,25-dihydroxyvitamin D [1,25(OH)_2_D] by suppressing the expression of 25-hydroxyvitamin D-1a-hydroxylase and increasing that of 25-hydroxyvitamin D-24-hydroxylase. FGF23 requires a-Klotho as well as FGF receptor to exert its effects.

FGF23 consists of 251 amino acids. Loss of function mutations in FGF23 cause familial tumoral calcinosis, which is characterized by hyperphosphatemia and increased levels of 1,25(OH)_2_D. Autosomal dominant hereditary hypophosphatemic rickets (ADHR) is caused by mutations in the cleavage RXXR motif, which prevent inactivation of FGF23. Excess of FGF23 action results in renal phosphate wasting and an inappropriately low level of serum 1,25(OH)_2_D. Recent studies have implicated the iron-deficiency in the late manifestation of ADHR. Tumor-induced osteomalacia is an acquired paraneoplastic syndrome of renal phosphate wasting caused by overproduction of FGF23 by tumors.

Among hereditary hypophosphatemic rickets/osteomalacia, X-linked hypophosphatemic rickets (XLH) is the most common form and is caused by deletion or inactivating mutations of the phosphate-regulating gene homologous to endopeptidase on X chromosome (PHEX). Autosomal recessive hypophosphatemic rickets type I and type II are caused by inactivating mutations of dentin matrix protein 1 (DMP1) and ectonucreotide pyrophosphatase/phosphodiesterase 1 (ENPP1), respectively. In these conditions, serum FGF23 levels are increased, which is responsible for the renal phosphate wasting and impaired vitamin D metabolism. FAM20C is a secreted kinase that phosphorylates extracellular proteins including DMP1. Recently, inactivating mutations in FAM20C have been reported in patients with increased levels of FGF23 and hypophosphatemia. PHEX, DMP1 and FAM20C as well as FGF23 are highly expressed in osteocytes, indicating the critical role of osteocytes in mineral metabolism. Hypophosphatemic rickets/osteomalacia caused by the increased bioactivity of FGF23 are classified into FGF23-mediated hypophosphatemic rickets/osteomalacia. Measurement of serum levels of FGF23 is useful for diagnosis of these conditions. Neutralization of FGF23 will be a potential treatment for XLH and other FGF23-mediated hypophosphatemic rickets/osteomalacia.

